# Mechanisms of isoniazid and rifampicin-induced liver injury and the effects of natural medicinal ingredients: A review

**DOI:** 10.3389/fphar.2022.1037814

**Published:** 2022-10-10

**Authors:** Xiuping Zhuang, Li Li, Tianyi Liu, Rui Zhang, Peimin Yang, Xin Wang, Long Dai

**Affiliations:** ^1^ School of Pharmacy, Binzhou Medical University, Yantai, China; ^2^ School of Pharmacy, Shandong University of Traditional Chinese Medicine, Jinan, China; ^3^ Department of Pediatrics, Affiliated Hospital of Shandong University of Traditional Chinese Medicine, Jinan, China; ^4^ Grade Three Laboratory of Traditional Chinese Medicine Preparation of the National Administration of Traditional Chinese Medicine, Affiliated Hospital of Shandong University of Traditional Chinese Medicine, Jinan, China

**Keywords:** isoniazid, rifampicin, liver injury, mechanism, natural medicinal ingredients, treatment

## Abstract

Isoniazid (INH) and rifampicin (RFP) are the first-line medications for tuberculosis treatment, and liver injury is the major adverse effect. Natural medicinal ingredients provide distinct benefits in alleviating patients’ symptoms, lowering the liver injury risk, delaying disease progression, and strengthening the body’s ability to heal. This paper summarises the recent research on the mechanisms of INH and RFP-induced liver injury and the effects of natural medicinal ingredients. It is believed that INH-induced liver injury may be attributed to oxidative stress, mitochondrial dysfunction, drug metabolic enzymes, protoporphyrin IX accumulation, endoplasmic reticulum stress, bile transport imbalance, and immune response. RFP-induced liver injury is mainly related to cholestasis, endoplasmic reticulum stress, and liver lipid accumulation. However, the combined effect of INH and RFP on liver injury risk is still uncertain. RFP can increase INH-induced hepatotoxicity by regulating the expression of drug-metabolizing enzymes and transporters. In contrast, INH can antagonize RFP-induced liver injury by reducing the total bilirubin level in the blood. *Sagittaria sagittifolia* polysaccharide, quercetin, gallic acid, and other natural medicinal ingredients play protective roles on INH and RFP-induced liver injury by enhancing the body’s antioxidant capacity, regulating metabolism, inhibiting cell apoptosis, and reducing the inflammatory response. There are still many gaps in the literature on INH and RFP-induced liver injury mechanisms and the effects of natural medicinal ingredients. Thus, further research should be carried out from the perspectives of liver injury phenotype, injury markers, *in vitro* and *in vivo* liver injury model construction, and liver-gut axis. This paper comprehensively reviewed the literature on mechanisms involved in INH and RFP-induced liver injury and the status of developing new drugs against INH and RFP-induced liver injury. In addition, this review also highlighted the uses and advantages of natural medicinal ingredients in treating drug-induced liver injury.

## 1 Introduction

Drug-induced liver injury (DILI) is liver damage caused by drugs, dietary supplements, and their metabolites, and the incidence rate is increasing yearly (Chakaya et al., 2021). DILI is the fifth leading cause of death due to liver diseases worldwide (Shen et al., 2019); thus, DILI received lots of attention. In China, the drugs that cause DILI mainly include non-steroidal anti-inflammatory, antitubercular, and anticancer drugs and drugs for metabolic diseases, among which the incidence of antitubercular drug-induced liver injury (ATB-ILI) is the highest (21.99%) (Zhang G, et al., 2020).

Isoniazid (INH) and rifampicin (RFP) are the first-line antitubercular drugs. The single or combined use of isoniazid and rifampicin can cause liver injury, leading to liver failure, accounting for 5%–22% of acute liver failure cases (Devarbhavi, et al., 2013). Natural medicinal ingredients have the characteristics of multi-level, multi-target, and multi-channel comprehensive regulation and have unique advantages in improving patients’ symptoms, reducing the risk of liver injury, delaying the progress of liver injury, and enhancing the repair ability of the body (Hong, et al., 2015). In recent years, natural medicinal ingredients have shown a good protective effect on liver injury caused by INH and RFP. This paper reviewed the molecular mechanisms of INH and RFP-induced liver injury and natural medicinal ingredients’ preventive and therapeutic effects on liver injury. We hope this review article will serve as an educational resource for researchers interested in developing new drugs against INH and RFP-induced liver injury.

## 2 The molecular mechanism involved in isoniazid-induced liver injury

The pathogenesis of isoniazid-induced liver injury (INH-ILI) has not been fully elucidated. The mechanisms of INH-ILI mainly involve oxidative stress, mitochondrial dysfunction, drug metabolic enzymes, protoporphyrin IX accumulation, endoplasmic reticulum stress, bile transport imbalance, and immune response.

### 2.1 Oxidative stress

**FIGURE 1:**
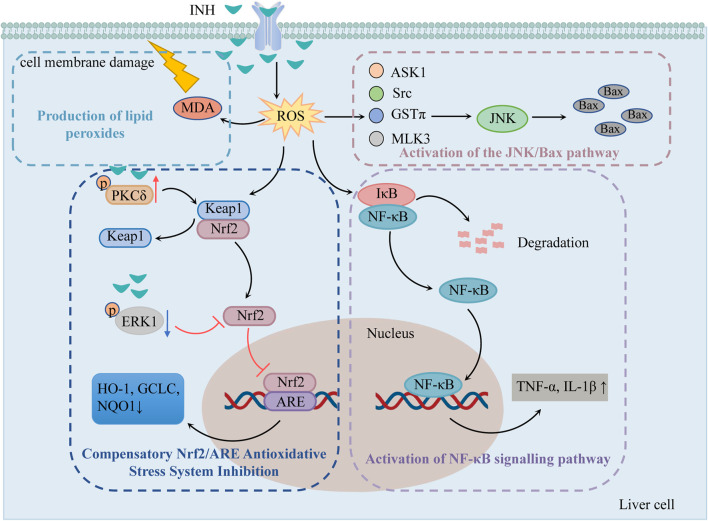
The mechanisms of oxidative stress injury caused by INH (Compensatory Nrf2/ARE antioxidative stress system inhibition, production of lipid peroxides, activation of NF-κB signaling pathway and the JNK/Bax pathway play important roles in INH-induced oxidative stress injury). Notes: INH, isoniazid; ROS, reactive oxygen species; MDA, malonic dialdehyde; Nrf2, nuclear erythroid 2-related factor 2; Keap1, kelch-like ECH-associated protein 1; PKCδ, protein kinase Cδ; ERK1, extracellular signal-regulated protein kinase 1; ARE, antioxidant response element; HO-1, heme oxygenase 1; GCLC, glutamine-l-cysteine ligase; NQO1, quinone oxidoreductase 1; NF-κB, nuclear factor-κB; IκB, NF-κB inhibitory protein; JNK, c-jun N-terminal kinase; ASK1, apoptosis signal-regulating kinase 1; Src, Src kinase; GSTπ, glutathione S-transferase π; MLK3, mixed-lineage protein kinase 3.

It has been suggested that the oxidative stress injury caused by INH is because of the dysregulated compensatory activation of the nuclear erythroid 2-related factor 2/antioxidant response element (Nrf2/ARE) antioxidant stress system and the reactive oxygen species (ROS) accumulation (Figure 1).

#### 2.1.1 Compensatory Nrf2/ARE antioxidative stress system inhibition

Nrf2/ARE is an important antioxidative stress signaling pathway in the body. When cells undergo oxidative stress, Nrf2 is uncoupled and translocated from Kelch-like ECH-associated protein 1 (Keap1) to the nucleus. Then, Nrf2 recognizes and binds to ARE and regulates many antioxidant mediators such as heme oxygenase 1 (HO-1), glutamine-l-cysteine ligase (GCLC), and quinone oxidoreductase 1 (NQO1), etc. Thereby improving the body’s ability to resist oxidative stress (Iranshahy et al., 2018). Previous studies have shown that 1) INH can induce protein kinase Cδ (PKCδ) phosphorylation to separate Nrf2 from Keap1, 2) INH can reduce the phosphorylation of extracellular signal-regulated protein kinase 1 (ERK1) and thus inhibits Nrf2 phosphorylation (Verma et al., 2015), and 3) reduces karyopherin β1 (KPNB1) level and thus blocks the entry of Nrf2 into the nucleus so that ARE can not be activated. All these mechanisms fail to activate Nrf2-mediated compensatory antioxidative mechanisms (Verma et al., 2018).

#### 2.1.2 Pathway of ROS accumulation-induced liver injury

As mentioned previously, the metabolite Hz generates a large amount of ROS *via* CYP2E1. Excessive ROS causes damage to DNA, lipids, and protein structure and produces 8-OH-deoxyguanosine and lipid peroxides such as malonic dialdehyde (MDA). MDA not only oxidizes the biofilm but also converts ROS into active substances and amplifies the effect of ROS through the chain reaction, causing cell membrane damage, which in turn leads to apoptosis and liver necrosis (Guo et al., 2015a).

ROS was found to stimulate nuclear factor-κB (NF-κB) inhibitory protein (IκB) to undergo phosphorylation and ubiquitination for degradation, exposing the nuclear localization sequence. The activated NF-κB translocates to the nucleus to initiate the gene transcription of TNF-α, IL-1β, and other factors, produces an inflammatory response, causes inflammatory infiltration of hepatocytes, and induces hepatic inflammatory damage (Shi, 2015).

ROS has been shown to activate c-jun N-terminal kinase (JNK) through apoptosis signal-regulating kinase 1 (ASK1), Src kinase, glutathione S-transferase π (GSTπ), and mixed-lineage protein kinase 3 (MLK3). The activated JNK can stimulate more ROS, leading to ROS burst, so that JNK is continuously activated and increases the expression of apoptotic protein Bax (Chen et al., 2018).

### 2.2 Mitochondrial dysfunction

**FIGURE 2:**
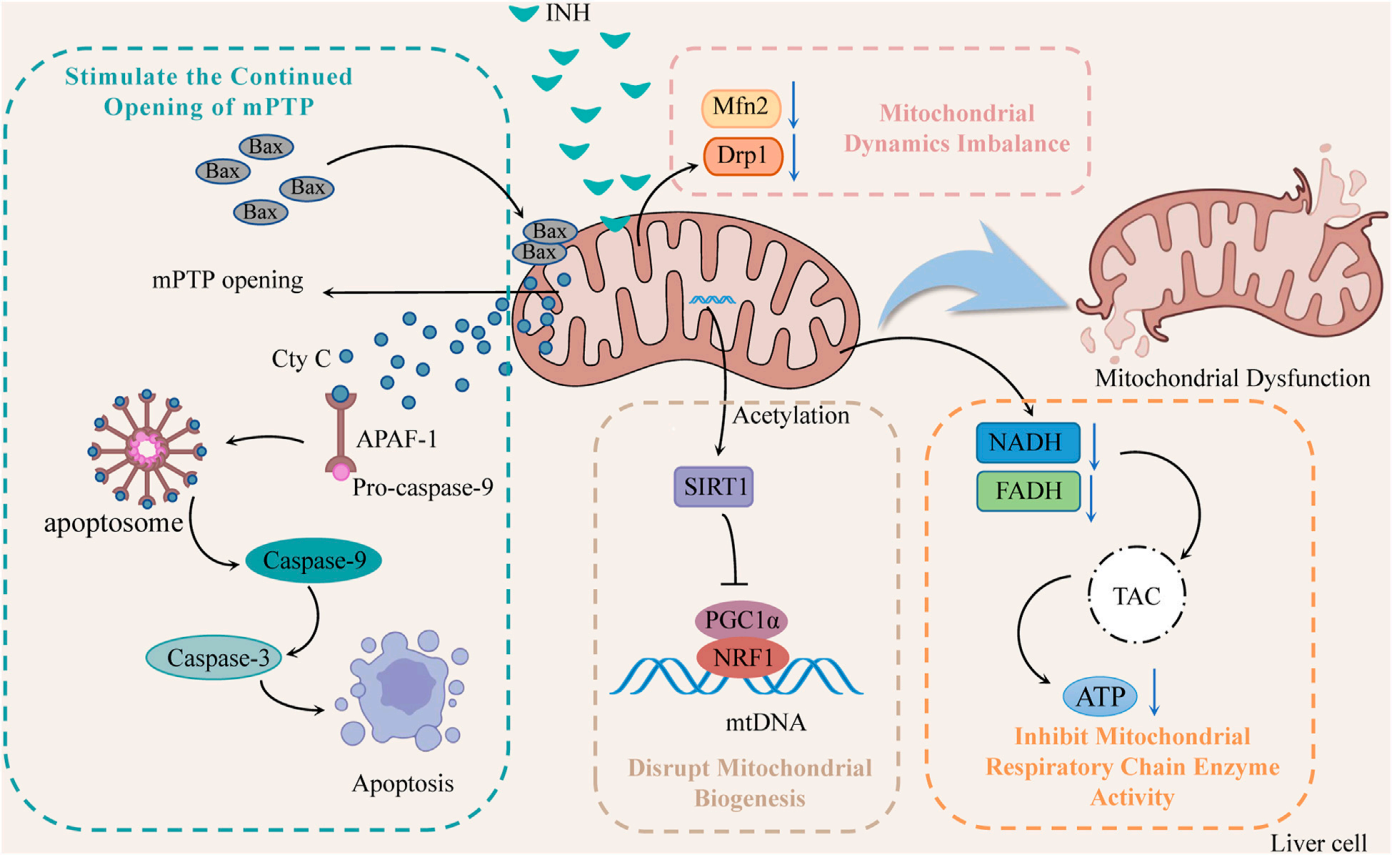
The mechanisms of INH-induced mitochondrial dysfunction (INH contributes to mitochondrial dysfunction by stimulating the continued opening of mPTP, disrupting mitochondrial biogenesis, inducing mitochondrial dynamics imbalance, and inhibiting mitochondrial respiratory chain enzyme activity). Notes: mPTP, mitochondrion permeability transition pore; Cyt C, cytochrome C; APAF-1, apoptotic protease activating factor-1; SIRT1, silent information regulator 1; PGC1α, peroxisome proliferator-activated receptor-gamma coactivator 1α; NRF1, nuclear respiratory factor 1; mtDNA, mitochondrial DNA; Mfn2, mitofusin 2; Drp1, dynamin-related protein 1; TCA, tricarboxylic acid cycle.

Mitochondria are one of the most sensitive organelles to various damages, leading to cellular energy depletion, apoptosis, and necrosis. INH reduces mitochondrial function by inhibiting the activity of mitochondrial respiratory chain enzymes, stimulating the continuous opening of mitochondrion permeability transition pore (mPTP), damaging mitochondrial biogenesis, and inducing mitochondrial dynamics imbalance (Figure 2).

#### 2.2.1 Inhibition of mitochondrial respiratory chain enzyme activity

INH significantly reduced the activity of NADH and FADH in the mitochondrial respiratory chain that transmits electrons and protons, which directly affected the tricarboxylic acid cycle in the mitochondria, reducing the ATP synthesis, and interfering with energy homeostasis (Lee et al., 2013; Lee et al., 2019; Wu et al., 2019). In addition, Zhu *et al.* found that INH and its acetylated product AcINH react with NAD under the catalysis of CD38 to form adducts INH-NAD and AcINH-NAD, which make NAD^+^ undergo structural changes, destroy its biological function, and lead to redox imbalance and energy homeostasis disorder (Zhu et al., 2020).

#### 2.2.2 Stimulate the continued opening of mPTP

Bax is overexpressed through the induction of INH, forming oligomers that transfer to the mitochondrial membrane, increase membrane permeability, and open mitochondrial ion channels and mPTP. Meanwhile, protons begin unrestricted movement across the inner membrane, resulting in oxidative phosphorylation uncoupling, mitochondrial membrane potential (ΔΨm) decreases, ATP synthesis decreases, and mitochondrial membrane lipid peroxidation increases rapidly (Lee et al., 2020). Mitochondria swell, eventually leading to mitochondria’s release of cytochrome C (Cyt C). Cyt C can combine with apoptotic protease activating factor-1 (APAF-1) and pro-caspase-9 to form apoptotic bodies and then cleave into caspase-9, thereby activating caspase-3 to induce apoptosis (Mu et al., 2020).

#### 2.2.3 Disrupt mitochondrial biogenesis

Mitochondrial biogenesis is mainly regulated by the mitochondrial biogenesis regulators silent information regulator 1 (SIRT1), peroxisome proliferator-activated receptor-gamma coactivator 1α (PGC1α) and nuclear respiratory factor 1 (NRF1) (Zheng et al., 2021). SIRT1 activates PGC1α through deacetylation, and activated PGC1α binds to NRF1 and promotes its transcription, thus driving mitochondrial biogenesis and maintaining dynamic balance within mitochondria (Mansouri et al., 2018). Studies (Zhang et al., 2017) have suggested that INH can increase SIRT1 acetylation, which subsequently fails to initiate PGC1 and NRF1 transcription, damage mitochondrial biogenesis, disrupt mitochondrial homeostasis, and induce ΔΨm reduction mitochondrial rupture and apoptosis.

#### 2.2.4 Induce mitochondrial dynamics imbalance

Mitochondria repair damaged mitochondria by constant division and fusion and maintain their normal morphology, function, and number. However, INH has been found to reduce the levels of mitofusin 2 (Mfn2) and dynamin-related protein 1 (Drp1), affect the normal division and fusion of mitochondria, make damaged mitochondria unable to repair, and decrease the number of mitochondria, resulting in the disturbance of the protective mitochondrial network, thereby increasing the release of Cyt C and initiating the apoptosis program (Li et al., 2019).

### 2.3 Drug metabolic enzyme

N-acetyltransferase 2 (NAT2), cytochrome P450 2E1 (CYP2E1), and glutathione S-transferase (GST) are involved in the metabolism of INH *in vivo*. These drug-metabolizing enzymes are highly polymorphic, which also contributes to the individual idiosyncratic nature of INH hepatotoxicity. However, its exact mechanism remains unknown.

#### 2.3.1 N-acetyltransferase 2

NAT2 is one of the important metabolic enzymes of INH, which can be divided into three types: slow, medium, and fast acetylation, based on the difference in acetylation rates among ethnic groups (Zahra et al., 2020). NAT2 can acetylate INH to acetyl isoniazid (AcINH) and hydrolyze it into acetyl hydrazine (AcHz), which is then oxidized to reactive metabolites (acetyldiazene, acetylonium ion, acetyl radical, and ketene), resulting in hepatotoxicity (Liu X et al., 2021). Therefore, NAT2 plays an irreplaceable role in the process of INH metabolism and hepatotoxicity. Studies have reported that slow acetylated patients are more likely to develop drug-induced hepatotoxicity, arise from the slow acetylated patients have slow acetylation of INH, and higher plasma drug concentrations, thereby increasing the risk of INH-ILI (Suvichapanich et al., 2018; Yang et al., 2019).

#### 2.3.2 Cytochrome P450 2E1

CYP2E1 is the central link between oxidative stress, ROS production, and hepatotoxic injury (Hassan et al., 2016). CYP2E1 oxidizes the AcHz produced by INH metabolism into reactive metabolites. These reactive metabolites covalently bind with cellular proteins and other macromolecules to cause lipid peroxidation and destroy the integrity of the hepatocyte membrane, and they also destroy the Ca^2+^-ATP enzyme system of the membrane, resulting in the imbalance of Ca^2+^ homeostasis in the internal and external environment of cells, eventually causing hepatocyte death (Boelsterli and Lee, 2014). Additionally, INH is partially hydrolyzed to hydrazine (Hz) *in vivo*, which mediates the generation of oxygen or superoxide free radicals to cause oxidative stress damage, which is also mediated *via* CYP2E1 (Wang C et al., 2016). CYP2E1 is highly polymorphic, in which the CYP2E1 *c1/c1* genotype had a higher CYP2E1 activity. A study showed that the patients with CYP2E1 *c1/c1* had a 4.57-fold higher risk of hepatotoxicity than other patients (Santos et al., 2019). Therefore, CYP2E1 *c1/c1* might be a risk factor for the increased incidence of hepatotoxicity in INH.

#### 2.3.3 Glutathione S-transferase

GST plays a protective role in the mechanism of liver detoxification, catalyzing glutathione reaction, facilitating solubilization, and excretion of toxic substrates. The *GSTM1* and *GSTT1* genotypes present polymorphisms by partial or total deletion (Li C. et al., 2013). Some studies (Chanhom et al., 2020) have shown that the *GSTM1* deletion type is significantly associated with the risk of INH-ILI in Southeast Asian populations. In contrast, the *GSTT1* deletion type and *GSTM1/GSTT1* double deletion type are not significantly associated with INH-ILI risk. It can be seen that the single deletion of *GSTM1* might be one of the factors that increase the hepatotoxicity of INH.

### 2.4 Protoporphyrin IX accumulation

Protoporphyrin IX (PPIX) is an intermediate in the biosynthesis of heme, which is mainly excreted through the biliary system. Excessive PPIX in bile can cause bile duct obstruction and cholestatic liver injury, promote protein oxidation, reduce protease activity, lead to mitochondrial dysfunction and heme generation disorder, and produce hepatotoxicity (Sachar et al., 2016). PPIX accumulation caused to INH has been proposed to be associated with delta-aminolevulinate synthase 1 (ALAS1) and ferrochelatase (FECH).

#### 2.4.1 Increase ALAS1 expression and promote PPIX synthesis

The INH metabolite Hz was found to act directly or indirectly on heme, causing N-alkylation and inactivation of cytochrome P450 enzymes, reducing heme content, activating the heme generation pathway in increased ALAS1 activity, and promoting the synthesis of PPIX (Lei et al., 2021).

#### 2.4.2 Reduce FECH expression and inhibit PPIX metabolism

Another metabolite of INH, pyridoxal isonicotinoyl hydrazone, was also found to chelate with Fe^3+^. Under low iron conditions, FECH will be degraded and cannot catalyze the conversion of ferrous ions and PPIX into heme (Brewer et al., 2019). This process changes the normal synthesis and metabolism of PPIX in the liver, leading to a large accumulation of PPIX in the liver.

### 2.5 Endoplasmic reticulum stress

When cells are disrupted to generate endoplasmic reticulum stress (ERS), misfolded proteins competitively bind the ER molecular chaperone glucose-regulated protein 78 (GRP78). The GRP78 interacts with the pathway protein kinase R-like endoplasmic reticulum kinase/activating transcription factor 4/C/EBP homologous protein (PERK/ATF4/CHOP). This interaction activates transcription factor 6 (ATF6), and inositol-requiring enzyme 1/X-box binding protein 1 (IRE1/XBP1) dissociate, causing phosphorylation and initiating an adaptive protective response (Lebeaupin et al., 2018). INH stimulated the overexpression of GRP78, ATF6, PERK, IRE1, and XBP1, which prevented properly initiating adaptive stress response to the endoplasmic reticulum (Zhang J et al., 2019). Strong or persistent ERS promoted CHOP expression, activated the caspase-12 apoptotic pathway, induced apoptosis, and triggered ERS damage.

### 2.6 Bile acid transport imbalance

Bile salt export pump (Bsep) and multidrug resistance-associated protein 2 (MRP2) are the transporters of bile acid. Bsep and MRP2 mediate the efflux of bile acid and bilirubin. The silent information regulator 1/farnesoid X receptor (SIRT1/FXR) pathway positively regulates Bsep and MRP2. Studies have shown that INH can inhibit SIRT1 deacetylation, thus inactivating FXR, causing Bsep and MRP2-mediated bile transport disorders and bile homeostasis imbalance in the liver, leading to cholestasis (Qu et al., 2018; Zhang W et al., 2020).

### 2.7 Immune response

Dysregulation of the liver immune environment is thought to play a critical role in the initiation and progression of DILI (Segovia-Zafra et al., 2021). One previous study had shown that lupus erythematosus developed in patients with INH-ILI (Shah et al., 2016). Lupus erythematosus is a well-known disease of the immune system, thus INH-ILI may be also related to the immune response. Metushi et al. (2014a) found that Th17 cells (inflammatory cells) were increased in patients with INH liver injury, suggesting that an immune-mediated mechanism of Th17 cells may be involved in the development of INH-ILI. A positive lymphocyte transformation test was found in mild cases of INH-ILI when patients’ lymphocytes were exposed to INH-modified proteins (Lei et al., 2021). Additionally, anti-INH and anti-CYP2E1 antibodies were detected in patients with liver failure, and IgG3 proved to be dominant among anti-INH antibodies (Metushi et al., 2014b). Results of these studies suggest that DILI may be related to immune response.

## 3 Molecular mechanisms of rifampicin-induced liver injury

**FIGURE 3:**
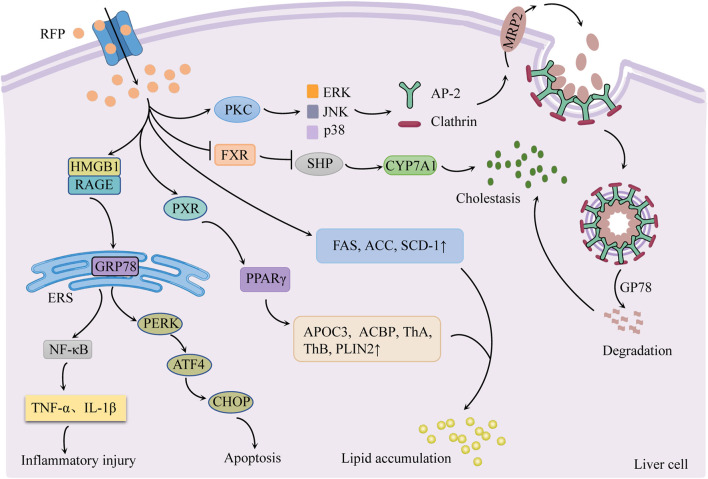
Schematic diagram of the molecular mechanisms of RFP-induced liver injury. Inhibition of FXR/SHP pathway promotes bile synthesis and activates PKC/ERK/JNK/p38 pathway to inhibit bile transport leading to cholestasis. Regulation of PERK/ATF4/CHOP and HMGB1/RAGE pathways to generate endoplasmic reticulum stress. RFP promotes hepatic fatty acid synthesis and absorption, respectively, by regulating hepatic fatty acid-related factors, resulting in the accumulation of hepatic lipids. Notes: RFP, rifampicin; FXR: farnesoid X receptor; SHP: small heterodimer partner; CYP7A1, cholesterol 7α hydroxylase; PKC, protein kinase C; ERK, extracellular signal-regulated kinases; JNK, c-jun N-terminal kinase; p38, p38 mitogen-activated protein kinases; AP-2, adaptin 2; GP78: E3 ubiquitin ligase; MRP2: multidrug resistance-associated protein 2; GRP78, glucose-regulated protein 78; HMGB1, high mobility group box 1 protein; RAGE: receptor for advanced glycation end products; PERK, protein kinase R-like endoplasmic reticulum kinase; ATF4, activating transcription factor 4; CHOP, C/EBP homologous protein; ERS, endoplasmic reticulum stress; NF-κB, nuclear factor-κB; FAS, fatty acid synthase; ACC, acetyl CoA carboxylase; SCD-1: stearoyl CoA desaturase 1; PXR: pregnane X receptor; PPAR-γ, peroxisome proliferator-activated receptor-γ; APOC3, apolipoprotein C-III, ACBP: acyl-CoA-binding protein; ThA, 3-ketoacyl-CoA thiolase A; ThB, 3-ketoacyl-CoA thiolase B; PLIN2, perilipin-2.

Cholestasis is currently proposed to be the main cause of RFP-induced liver injury. RFP is also involved in endoplasmic reticulum stress and hepatic lipid accumulation (Figure 3).

### 3.1 Cholestasis

Cholestasis is the main cause of RFP-induced liver injury, characterized by increased bile acid synthesis and decreased bile acid transport.

#### 3.1.1 Regulation of FXR/SHP pathway to promote bile synthesis

Cholesterol 7α hydroxylase (CYP7A1) is a rate-limiting enzyme in bile acid synthesis in hepatocytes. Its activity is regulated by the negative feedback of the farnesoid X receptor/small heterodimer partner (FXR/SHP) pathway (Sanoh et al., 2019). There was evidence (Xu et al., 2016) that RFP suppresses the FXR/SHP pathway, increases CYP7A1 mRNA expression, and promotes bile acid synthesis in hepatocytes. This sequence of events causes the liver to accumulate hydrophobic bile acids.

#### 3.1.2 Activation of the PKC/ERK/JNK/p38 pathway to inhibit bile transport

Xu et al. (2020) found that RFP could activate protein kinase C/extracellular signal-regulated kinases/c-jun N-terminal kinase/p38 mitogen-activated protein kinases (PKC/ERK/JNK/p38) signaling pathway. The PKC/ERK/JNK/p38 pathway stimulates junction protein adaptin 2 (AP-2) and clathrin to mediate MRP2 endocytosis and reduce MRP2 quantity. Simultaneously, RFP could increase E3 ubiquitin ligase GP78 activity, induce MRP2 ubiquitination degradation, cause bile transport dysfunction, and lead to eventual cholestasis (Chen et al., 2022).

### 3.2 Endoplasmic reticulum stress

RFP mainly regulates PERK/ATF4/CHOP pathway and high mobility group box 1 protein/receptor for advanced glycation end products (HMGB1/RAGE) pathway to produce ERS response, resulting in apoptosis and inflammatory response.

#### 3.2.1 Activation of the PERK/ATF4/CHOP pathway to induce apoptosis

Zhang *et al.* reported that normal L02 cells treated with RFP (200 μmol/L for 48 h) had enhanced mRNA expression of GRP78, PERK, ATF4, and CHOP (Zhang G et al., 2016). Thus, it may be interpreted that RFP produces ERS response by activating the PERK/ATF4/CHOP pathway. Long-term or sustained ERS phosphorylates eukaryotic initiation factor 2α (eIF2α) and promotes the transcription and translation of ATF4, thus increasing CHOP activity and inducing apoptosis (Chen et al., 2014).

#### 3.2.2 Activation of the HMGB1/RAGE pathway to trigger an inflammatory response

It was found that RFP induced HMGB1 to bind to RAGE on the surface of hepatic stellate cells. This binding activated the downstream signal factors and promoted the ERS signal transduction, leading to ERS generation. ERS can trigger inflammatory reactions *via* NF-κB activation and tumor necrosis factor release. At the same time, inflammatory factors act as agonists on NF-κB, forming a positive feedback regulation loop of NF-κB, which aggravates liver lesions (Lu, 2020).

### 3.3 Hepatic lipids accumulation

RFP promotes the synthesis and absorption of hepatic fatty acids by regulating hepatic fatty acid-related factors, resulting in the accumulation of hepatic lipids.

#### 3.3.1 Increase hepatic fatty acid synthesis

One study (Huang et al., 2016) showed that RFP increased the activities of fatty acid synthase (FAS), acetyl CoA carboxylase (ACC), and stearoyl CoA desaturase 1 (SCD-1) in the liver, which promotes hepatic fatty acid synthesis and elevated sliver triglycerides (TGs).

#### 3.3.2 Promote liver fatty acid absorption

Peroxisome proliferator-activated receptor-γ (PPAR-γ) is a ligand-activated transcription factor that promotes fatty acid absorption in the liver. The pregnane X receptor (PXR) is the main nuclear receptor regulating the expression of xenobiotic-metabolizing enzymes and is highly expressed in the liver and intestine (Barretto et al., 2019). RFP was found to enhance the expression of PPAR-γ and its downstream apolipoprotein C-III (APOC3), acyl-CoA-binding protein (ACBP), 3-ketoacyl-CoA thiolase A (ThA) and B (ThB) and perilipin-2 (PLIN2) by activating PXR in the liver to increase fatty acid uptake and lipid content in liver circulation, resulting in lipid accumulation (Kim et al., 2017).

## 4 Molecular mechanisms involved in the liver injury caused by isoniazid and rifampin

The combined effects of INH and RFP on liver injury are variable among different studies. Most studies suggested that RFP could increase INH-induced liver injury by modulating the drug metabolism and transport enzyme expression *via* agonizing PXR activity (Zhang et al., 2019a; Wang et al., 2019). Nevertheless, few studies support that INH reduces RFP-induced liver injury by reducing serum alanine transaminase (ALT) levels and total bilirubin (TBIL) (Xiang et al., 2019).

### 4.1 RFP increases the INH-Induced liver injury by inducing CYP

RFP induces the CYP3A4 expression *via* PXR activation, and CYP3A4 deacetylates RFP (Pan et al., 2020). Unlike INH, the metabolites of RFP are mostly considered nontoxic. However, in the presence of INH, RFP donates the acetyl group to INH. The acylated INH undergoes accelerated metabolism to AcHz (Chen J et al., 2016; Combrink et al., 2020), and thus, RFP significantly increases INH-induced liver injury. In addition, RFP induces the CYP2E1 expression *via* PXR activation, and thereby increasing the hydrazine production and aggravating the damage caused by INH-induced oxidative stress.

### 4.2 RFP promotes PPIX accumulation to aggravate the liver injury

RFP upregulated ALAS1 levels *via* PXR activation, working with INH to substantially increase PPIX synthesis (Li F. et al., 2013). INH metabolites inhibit FECH and thus block the binding of PPIX with ferrous ions to form heme (Brewer et al., 2020; Wang C et al., 2020). The increased heme causes further accumulation of PPIX and cholestatic liver injury.

Some studies (Xiang et al., 2019) claimed that INH could antagonize RFP-induced liver injury by reducing the ALT and TBIL levels. However, the mechanism is unclear. As illustrated in Table 1, there are large differences in the evaluation of INH and RFP-induced liver injuries, such as dose, dosage, administration time, and the degree of liver injury, which may be the underlying reasons for these different results.

**TABLE 1:** INH and RFP liver injury model and summary of research results.

Rat or mouse strains	Modelling dose and time	Detection index	Research results
SPF grade Kunming mice	90 mg/kg INH, gavaged for 3, 5, 7, 10, and 15 days	ALT, AST↑; MDA↑, SOD↓; NF-κB↑, TNF-α mRNA and protein↑, IκB mRNA and protein↓	INH down-regulate IκB, activate the NF-κB pathway, upregulates proinflammatory factor TNF-α, and induces inflammatory reactions Shi, (2015)
C57B/6J mice	100 mg/kg INH, gavaged twice a day, and killed on the 5th and 14th day, respectively	Liver fatty lesions↑; ALT, AST↑; LDH, SDH↑; GSSG, GSSG/GSH↑, GSH↓; NADH, FADH, ATP, ΔΨm↓	INH can damage liver mitochondria and lead to hepatic steatosis through oxidative stress Wu et al. (2019)
Male FVB/NJ mice	Drinking water containing 400 mg/L INH was administered continuously for 3, 7, 14, 28 days, and drinking water containing 100, 200, 400 mg/L INH for 14 days	PPIX in the liver↑, PPIX levels in bone marrow cells, serum or red blood cells were unchanged; FECH protein↓; ALAS1 protein↑; All indexes are time-and dose-dependent	INH induce ALAS1 protein expression in both a time-dependent and dose-dependent manner and decrease FECH protein expression, leading to PPIX accumulation in the liver Sachar et al. 2016)
Male Wistar rats	50, 200, 350 mg/kg INH and continuous gavaged for 7 days	High dose group:ALT, AST,TBA,DBIL, TBIL↑; Medium dose group; ALT, DBIL and TBIL↑: High and medium-dose group: MRP2 and Bsep proteins↓; SIRT1 and FXR proteins↓	INH inhibit the expression of the bile acid transporters Bsep and MRP2 by blocking the SIRT1/FXR pathway, causing cholestatic liver injury Qu et al. (2018)
SPF grade Kunming mice	180 mg/kg RFP continuous gavaged for 30 days and 90 mg/kg RFP continuous gavaged for 90 days	180 mg/kg RFP 30 days: ALT↑; CYP7A1, FXR, SHP genes↑, Bsep protein and gene↓. 90 mg/kg RFP 90 days:CYP7A1 protein and gene↑	RFP promote bile synthesis and suppresses excretion by regulating the expression of CYP7A1 and Bsep, causing cholestatic liver injury (Xu et al., 2016)
SPF grade C57BL/6 mouse	200 mg/kg RFP regularly gavaged for 7 days	TBIL, DBIL, ALP, ALT↑; TBA, MDA↑; SOD↑; intracytoplasmic HMGB1 and AC-HMGB1↑; RAGE, NF-κB proteins↑	RFP is involved in the development process of liver injury through the activation of the HMGB1/RAGE/NF-κB signaling pathway Lu, (2020)
Male CD-1 mice	200 mg/kg RFP and gavaged for 3 days, 1 week and 4 weeks	ALT, TG↑; FAS, ACC, SCD-1 mRNA↑; PPARγ gene and protein↑; PXR protein↑	RFP can increase hepatic fat synthesis and activate PXR to upregulate PPARγ expression to increase fatty acid uptake and induce hepatic fat accumulation (Huang et al., 2016)
Male ICR mice	177, 442.5 mg/kg RFP continuous gavaged for 2 weeks	Liver weight↑; ALT, AST, ALP↑; PPARγ protein↑; APOC3, ACBP, ThA, ThB and PLIN2↑	A PPARγ signaling pathway is closely related to RFP-induced liver injury Kim et al. (2017)
SPF grade male Kunming mice	Continuous gavaged: 150 mg/kg INH, 300 mg/kg RFP, or 300 mg/kg+300 mg/kg INH/RFP for 1–3 weeks	Single gavage: TBIL and DBIL↑. Continuous gavage for 1 week: ALT, AST, TBIL↑; and more severe in INH/RFP group; 3 weeks: ALT and TBIL↓ in INH/RFP group	RFP is the dominant factor causing liver injury in mice, and combination with INH antagonises the trend of RFP liver toxicity Xiang et al. (2019)
Male small BALB/c mice	50 mg/kg INH and 100 mg/kg RFP gavaged for 6 days weekly for 4–24 weeks	ALT, TG↑; SOD, GSH-Px and CAT after 12 weeks↓; liver fibrosis↑; HSC quantity↑; NOX, CYP2E1 activity↑; Bcl-2 protein↓, Cyt C in the cytosol↑, Bax protein↑, caspase-3 activity↑	Long-term use of INH and RFP trigger NO X-mediated oxidative stress, induces hepatocyte apoptosis and activates HSC, leading to liver fibrosis Biswas et al. (2020)
SPF grade SD rats	50 mg/kg INH and 50 mg/kg RFP continuous gavaged for 28 days	IL-33, IL-18, IL-1↑; NLRP3, ASC, Caspase-1 protein↑; SOD, CAT, GSH and GSH-Px↓; CYP2E1 activity↑	INH and RFP reduce drug-metabolising activity enzymes by regulating the NLRP3 inflammasome and inducing inflammatory responses and oxidative stress in hepatocytes Su et al. (2021)
Female ICR mice	Low-dose group: 50 mg/kg INH, 100 mg/kg RFP, 50 mg/kg+100 mg/kg INH/RFP continuous gavaged for 14 days. High-dose group: 100 mg/kg INH, 200 mg/kg RFP, 100 mg/kg+200 mg/kg INH/RFP, gavaged for 14 days	Ntcp, Bsep protein↓; ALT, AST, ALP, TBIL, DBIL, TBA↑; MDA↑; The higher dose group was stronger than the lower dose group, the INH/RFP group was stronger than the INH group and the RFP group	INH/RFP leads to bile acid accumulation and induces liver injury by downregulating bile acid transporter Ntcp and Bsep expression Guo et al. (2015b)

Notes: INH, isoniazid; RFP, rifampicin; MDA, malonic dialdehyde; SOD, superoxide dismutase; NF-κB, nuclear factor-κB; IκB, NF-κB, inhibitory protein; LDH, lactate dehydrogenase; SDH, sorbitoldehydrogenase; GSSG, oxidized glutathione; GSH, glutathione; PPIX, protoporphyrin IX; ALAS1, delta-aminolevulinate synthase 1; FECH, ferrochelatase; TBA, total bile acid; DBIL, direct bilirubin; TBIL, total bilirubin; Bsep, Bile salt export pump; MRP2, multidrug resistance-associated protein 2; SIRT1, silent information regulator 1; FXR, farnesoid X receptor; FXR, farnesoid X receptor; SHP, small heterodimer partner; CYP7A1, cholesterol 7α hydroxylase; HMGB1, high mobility group box 1 protein; AC-HMGB1, Acetyl high mobility group box 1 protein; RAGE, receptor for advanced glycation end products; TG, triglyceride; FAS, fatty acid synthase; ACC, acetyl CoA carboxylase; SCD-1, stearoyl CoA desaturase 1; PXR, pregnane X receptor; PPAR-γ, peroxisome proliferator-activated receptor-γ; APOC3, apolipoprotein C-III; ACBP, acyl-CoA-binding protein; ThA, 3-ketoacyl-CoA, thiolase A; ThB: 3-ketoacyl-CoA, thiolase B; PLIN2, perilipin-2; GSH-Px, glutathione peroxidase; CAT, catalase; HSC, hepatic stellate cell; NOX, NADPH, oxidase; CYP2E1, cytochrome P2E1; Cyt C, cytochrome C; NLRP3, NLR, pyrin domain containing 3; ASC, adaptor protein apoptosis speck-like protein containing CARD; Ntcp, sodium taurocholate cotransporting polypeptide.

## 5 Protective effect of natural medicinal ingredients on isoniazid and rifampicin-induced liver injury

### 5.1 Polysaccharides

Natural polysaccharides are widely found in the plant kingdom. Natural medicinal ingredients-containing polysaccharides mainly protect the liver by enhancing the antioxidation ability, regulating metabolism, inhibiting apoptosis, reducing inflammation, etc.

*Sagittaria sagittifolia* polysaccharide is refined from the water extract of *Sagittaria Sagittifolia L*. It is the effective ingredient of *Sagittaria Sagittifolia L.* *Sagittaria sagittifolia* polysaccharide can increase the antioxidant capacity of the body by activating the compensatory Nrf2/ARE antioxidant stress system, inhibit CYP2E1 and CYP3A4, reduce hepatotoxicity, inhibit hepatocyte apoptosis, improve cell survival rate, regulate metabolic pathway and restore homeostasis to protect liver injury caused by combined application of INH and RFP. The protective mechanisms of *Sagittaria sagittifolia* polysaccharide on INH and RFP-induced liver injury are shown in Table 2.

**TABLE 2:** The protective mechanisms of *Sagittaria sagittifolia* polysaccharide on INH and RFP-induced liver injury.

Dose and model	Dosage and time of administration	Changes in indexes after administration	Mechanisms of action
100 mg/kg INH +100 mg/kg RFP. Male BALB/c mice	The drug was administered at 200, 400 and 800 mg/kg for 30 days	ALT, AST↓; LDH↓; GSH, SOD, CAT ↑; MDA ↓; CYP2E1 and CYP3A4 proteins and genes↓; Intranuclear Nrf2, HO-1, GCLC proteins and genes↑	Activate Nrf2 and its downstream associate antioxidant enzymes, and inhibit CYP2E1 and CYP3A4 activity Wang et al. (2018a)
0.1 mg/ml INH +0.2 mg/ml RFP HepG2 cells	0.15, 1, 1.5, 2, 2.5 mg/ml for 24 h	CYP2E1 and CYP3A4 enzyme content, genes, and proteins↓	Inhibition of INH and RFP combined induction of CYP2E1 and CYP3A4 to reduce liver injury Wang et al. (2018b)
0.1 mg/ml INH +0.2 mg/ml RFP HepG2 cells	0.125, 0.25, 0.5, 1, 2 mg/ml for 24 h	ALT, AST↓; HO-1, GCLC proteins and genes↑, and increased significantly at 0.5 and 1 mg/ml	Enhance HO-1 and GCLC enzyme activities, inhibit oxidative stress in HepG2 cells Liu H S et al. (2021)
1.1 mg/ml INH +0.2 mg/ml RFP HepG2 cells	0.125, 0.25, 0.5, 1, 2 mg/ml for 24 h	ALT, AST↓; LDH↓; Nrf2, HO-1, GCLC proteins and genes↑, Keap1 proteins and genes↓; CYP2E1, CYP3A4 proteins, and genes↓; Bcl-2↑; Bax, Caspase-3, Caspase-9↓; Bcl-2/Bax↑	Activate Nrf2/ARE signal pathway, and inhibit CYP2E1 and CYP3A4 enzyme expression by inhibiting hepatocyte apoptosis Wang. (2018)
0.1 g/kg INH + 0.1 g/kg RFP. Male BALB/c mice	8 g/kg for 30 days	ALT and AST↓; liver histopathology↓; inflammatory infiltrate↓; altered metabolic pathways and related markers such as TCA cycle, ornithine metabolism, taurine metabolism and amino acid metabolism; nuclear Nrf2 and HO-1 proteins↑	Adjust the TCA cycle, ornithine cycle, branched-chain amino acid cycle, fatty acid metabolism and other ways, improve adipose lesions, and regulate energy metabolism Ke et al. (2018); Ke. (2019)
0.1 g/kg INH + 0.1 g/kg RFP. Male BALB/c mice. 1.1 mg/ml INH +0.2 mg/ml RFP HepG2 cells	BALB/c mice; 0.2, 0.4, 0.8 g/kg for 30 days. HepG2 cells: 0.125, 0.25, 0.5, 1, 2 mg/ml for 24 h	Nrf2↑ in the nucleus, HO-1↑ in the cytoplasm, Keap1↓; Bcl-2↑, Bax↓	Regulate Nrf2/Keap1 signaling pathway and apoptosis genes Bcl-2 and Bax to reduce apoptosis Liu et al. (2022)

Notes: INH, isoniazid; RFP: rifampicin; SOD, superoxide dismutase; LDH, lactate dehydrogenase; SDH, sorbitoldehydrogenase; GSH, glutathione; MDA, malonic dialdehyde; CAT, catalase; CYP2E1, cytochrome P2E1; CYP3A4, cytochrome P3A4; Nrf2, nuclear erythroid 2-related factor 2; Keap1, Kelch-like ECH-associated protein 1; HO-1, heme oxygenase 1; GCLC, glutamine-l-cysteine ligase; TCA, tricarboxylic acid cycle.

Prunella vulgaris sulfated polysaccharides have antitumour and antifibrotic effects. Wang *et al.* reported that polysaccharides treatment (100 mg/kg for 14 days) in SPF male C57BL/6 mice protected them from liver injury (Wang R et al., 2021). The treatment causes the regeneration of hepatocytes and decreases inflammatory cell infiltration. The treatment reduced the serum AST and ALT levels and lipid peroxide content in liver tissue. The treatment has improved the SOD activity and inhibited the expression (genes and proteins) of IL-6 and TNF-α (inflammatory factors). It also attenuated the INH-ILI by antioxidative stress and reduced the inflammatory response.

In Wistar male rats, seaweed polysaccharides were protective against INH and RFP-induced liver injury. They reduced the MDA content, increased the GSH activity, slowed down the pathological changes of liver tissue, protected the liver from oxidative damage, upregulated the expression of bile transporter sodium taurocholate co-transporting polypeptide (Ntcp), accelerated the bile salt circulation, promoted the bile excretion, reduced the bile salt accumulation (Fang et al., 2017).

### 5.2 Flavonoids

Quercetin has anti-inflammatory, antioxidant, and anticancer effects. It is mainly present in Folium Mori, Flos Sophorae Immaturus, Fructus Crataegi, etc. It regulates INH-ILI through multiple pathways. Quercetin 1) inhibits oxidative stress, 2) reduces ROS accumulation, and 3) repairs mitochondrial function by regulating the Nrf2-related signaling pathway. It also 1) reduces apoptosis and 2) improves cell survival by inhibiting ROS/Caspase-3, ROS/JNK, and SIRT1/ERK apoptosis pathways. In addition, quercetin can also inhibit NLRP3 inflammatory bodies and reduce the inflammatory response to exert its anti-INH-ILI effect. The protective mechanisms of quercetin on INH-ILI are shown in Table 3.

**TABLE 3:** The protective mechanisms of quercetin on INH-ILI.

Dose and model	Dosage and time of drug administration	Changes of indexes after administration	Mechanisms of action
10 mmol/L INH. L02 cells	25, 50 μmol/L for 24 h	Cell DNA tailing↓; cell mitochondrial ROS level↓; ΔΨm↑; The effect of the high dose group was stronger than that of the low dose group	Inhibition of ROS-mediated mitochondrial damage reduces cellular mitochondrial ROS generation and enhances cellular mitochondrial membrane potential Chen T Y et al. (2016)
10 mmol/L INH. L02 cells	25, 50 μmol/L for 24 h	Cell survival rate↑; cell apoptosis rate↓; LDH activity↓; mitochondrial ROS level↓; ΔΨm↑; Caspase-3 protein↓	Inhibition of ROS released, improvement of mitochondrial function, inhibition of ROS/Caspase-3 signaling pathwayChen et al. (2019)
10 mmol/L INH. L02 cells	25, 50 μmol/L for 24 h	Cell survival rate↑; cell mitochondrial ROS level↓; MDA↓; GSH, SOD↑; HO-1 protein in the cytoplasm and Nrf2 protein in the nucleus↑	Regulate the Nrf2/ARE signaling pathway and inhibit mitochondrial oxidative damage Chen et al. (2021a)
10 mmol/L INH. L02 cells	50 μmol/L for 24 h	Cell survival rate↑; cell apoptosis rate↓; LDH activity↓; relative Caspase-3 activity ↓; mitochondrial ROS ↓; p-JNK protein↓	Inhibit ROS/JNK pathway to decrease apoptosis Chen et al. (2021b)
52 mmol/L INH. HepG2 cells	0.1, 1 mg/L for 24 h	Cell survival rate↑; ALT, AST↓; GSH, SOD↑; Bcl-2↑; Bax, Caspase-3, Caspase-9↓; SIRT1 and ERK phosphorylation↑; apoptosis rate↓; ΔΨm↑	Inhibition of mitochondrial oxidative stress, activation of SIRT1/ERK pathway and inhibition of apoptosis Zhang et al. (2019b)
300 mg/kg INH. Male SD rats; 50 mmol/L INH, L02 cells	Male SD rats: 50, 100 mg/kg for 10 days; L02 cells: 5 and 10 μmol/L for 24 and 48 h	ALT, AST↓; ASC, NLRP3 inflammasome, Caspase-1 and IL-1 proteins↓; SIRT1 protein↑; Caspase-3, Bax/Bcl2↓; cell apoptosis rate↓; ΔΨm↑	Activate SIRT1 signaling pathway, inhibite the activation of NLRP3 inflammatory bodies to decrease cell apoptosis Zhang and Lu. (2020)

Notes: INH, isoniazid; RFP, rifampicin; SOD, superoxide dismutase; LDH, lactate dehydrogenase; SDH, sorbitoldehydrogenase; GSH, glutathione; MDA, malonic dialdehyde; ROS, reactive oxygen species; ΔΨm, mitochondrial membrane potential; Nrf2, nuclear erythroid 2-related factor 2; ARE, antioxidant response element; HO-1, heme oxygenase 1; JNK, c-jun N-terminal kinase; SIRT1, silent information regulator 1; ERK, extracellular signal-regulated kinases; NLRP3, NLR, pyrin domain containing 3; ASC, adaptor protein apoptosis speck-like protein containing CARD.

Total flavonoids from *Polygonum perfoliatum L* (150, 300, and 600 mg/kg) can reduce lipid peroxide content, increase SOD activity, promote the expression of Bcl-2, Nrf2 and HO-1, and inhibit Bax expression. It alleviates oxidative stress and apoptosis in INH and RFP-induced liver injury in mice by activating the Nrf2/ARE signaling pathway (Wang X X et al., 2021). Another study (Xi et al., 2017) showed that total flavonoids blocked Fas-mediated apoptosis, inhibited TNF-α, IL-1, and IL-6, reduced inflammatory response and exerted liver protection.

Hesperidin is a flavonoid glycoside abundant in *Citri Reticulatae Pericarpium* and *Aurantii Fructus Immaturus.* Oxidative stress generated by INH and RFP treatment can deplete reduced glutathione and increase oxidized glutathione levels in the liver. Hesperidin enhances the reduced glutathione activity in the liver, further upregulates the expression of MRP2, promotes oxidized glutathione’s excretion into the bile, maintains the redox balance in hepatocytes, and reduces oxidative stress (Zhang W et al., 2016).

Naringin from *Rhizoma Drynariae*, *Fructus Aurantii*, has shown antioxidant and free radical scavenging activities. Wang *et al.* reported the protective effect of naringin in INH/RFP liver injury in male BALB/c mice. Naringenin reduced the MDA content, increased the GSH and SOD activities, inhibited oxidative stress, regulated the pro-apoptotic protein Bax and the anti-apoptotic protein Bcl-2, and suppressed the activity of caspase-3 and reduced apoptosis (Wang P et al., 2016).

### 5.3 Polyphenols

Gallic acid mainly exists in *Galla Chinensis*, showing free radical scavenging, antioxidant, antitumor, and anti-inflammatory activities. Sanjay *et al.* reported the protective effect of gallic acid in INH and RFP-induced liver injury in Wistar rats (Sanjay et al., 2021). It reversed the elevated AST, ALT, and ALP levels, activated the Nrf2 pathway, upregulated the gene expression of endogenous antioxidants (SOD, CAT, GSH-Px, and GSH), decreased the ROS accumulation, and inhibited Toll-like receptor 4 (TLR-4), reduced the levels of inflammatory mediators HMGB-1 and Interferon-γ (IFN-γ), inhibited the NF-κB activation, down-regulated the IL-1β and NOS2 expression, and reduced the inflammatory responses.

Curcumin is extracted from the rhizomes of turmeric. An *in vivo* and *in vitro* study reported that it enhanced the FECH expression, reduced PPIX level in the liver, and induced the breast cancer resistance protein (BCRP) in INH and RFP-induced liver injury model. The BCRP induction accelerates the PPIX efflux and decreases the PPIS accumulation in hepatocytes (He et al., 2017).

### 5.4 Others

The protective effect of ursolic acid (from *Hippophae Fructus*) on INH and RFP-induced liver injury is mediated *via* the regulation of the NLRP3/caspase-1 pathway. The *in vitro* liver injury model was established by treating HL-7702 cells with INH (120 g/ml) and RFP (240 g/ml). Treatment with ursolic acid (0.018, 0.18, and 1.8 μmol/L) increased the cell survival rate, elevated the SOD level, decreased the MDA level, TNF-α, and IL-1β, suppressed the NLRP3 activation, reduced pro-caspase-1 and caspase-1 protein levels, inhibited the apoptosis and promoted the cell proliferation (Dou and Yu, 2021).

Tanshinone IIA is a diterpene quinone compound. Yang et al. (2020) used L02, HEK293 cells, and C57BL/6 mice to study the protective effect of Tanshinone IIA in RFP-induced liver injury. Tanshinone IIA induced the expression of 10–11 translocation methylcytosine dioxygenase 2 (TET2) and mediated Nrf2 demethylation. Activated Nrf2 promotes Bsep and Ntcp transcription by recognizing and binding to muscle aponeurosis fibrosarcoma recognition element (MARE). The compound promoted bile transport and bile acid efflux, while Nrf2 knockout induces the elimination of bile transporters. It is suggested that the protective effect of tanshinone IIA on RFP-induced cholestatic liver injury is *via* Nrf2 activation.

Schizandrin B is a biphenyclooctene lignan from *Schisandrae Chinensis Fructus*. It has shown antioxidant, free radical scavenging, anti-inflammatory, and anti-apoptotic activities. RFP promotes apoptosis in L02 cells, decreases cell survival rate, and increases the gene and protein expression of GRP78, PERK, ATF4, CHOP, ATF6, p-IRE1, and XBP-1. Schisandrin B improved the hepatocyte survival rate in a dose- and time-dependent manner. It reduced the apoptosis rate, reversed the protein and gene expression levels of ERS-related signaling pathways, and mitigated the L02 cell damage induced by RFP (Cheng et al., 2020).

Magnesium isoglycyrrhizinate (MgIG), the fourth-generation glycyrrhizic acid preparation, is more effective in preventing ATB-ILI than other glycyrrhizic acid preparations, which is possibly due to the higher clearance rate of 18α-glycyrrhizin *in vivo* than 18β-glycyrrhizin. MgIG has a lower incidence of adverse reactions and higher safety than 18β-glycyrrhizin (Xu et al., 2013), which has been verified in 97 randomized controlled trials (Gong et al., 2022a). Additionally, the studies *in vivo* found that MgIG effectively ameliorated ATB-ILI by restoring *lactobacillus* abundance, enhancing intestinal barrier function, and further inhibiting the lipopolysaccharide-activated TLRs/NF-κB signaling pathway (Gong et al., 2022b).

In addition, N-trans-Caffeoyldopamine, Sinomenine, Yulangsan polysaccharide, and others have a certain protective effect on INH and RFP-induced liver injury. The protective activities of these compounds are summarised in Table 4.

**TABLE 4:** Study on other natural medicinal ingredients against INH and RFP-induced liver injury.

Ingredient name	Chinese medicine source	Detection indicators	Mechanisms of action
N-trans-Caffeoyldopamine	*Capsicum annuum, Lycium chinense*	ALT, AST↓; SOD, GSH↑, MDA↓; CYP2E1 mRNA↓	Modulate CYP2E1 mRNA expression, reduce free radicals and ROS, and improve antioxidant capacity Wu et al. (2015)
Sinomenine	*Caulis Sinomenii*	ALT, AST, TG↓; SOD, GSH↑, MDA↓	Reduce oxygen free radicals, and inhibit lipid peroxidation Yu et al. (2021)
Yulangsan polysaccharide	*Millettia pulchra*	Liver index↓; ALT, AST↓; SOD, GSH-Px, GSH↑, MDA↓	Radical scavenging action and antioxidant activity Dong et al. (2014)
Resveratrol	*Fructus Mori, Polygoni Cuspidati Rhizoma et Radix*	ALT, AST↓; CAT, GSH↑; TNF-α, IL-12p70, IL-10↓; SIRT1↑, PPAR-γ↓, PGC1α↓	Modulation of SIRT1 and PPAR-γ/PGC1α expression Nicoletti et al. (2014)
Bicyclol	Schisandrae Chinensis Fructus	ALT, AST, TBIL↓; SOD, GSH-Px, GSH, CAT↑, MDA↓; TNF-α, IL-1β↓; CYP2E1↓	attenuate oxidative stress, suppress cytokine overexpression, modulate CYP2E1 Liu et al. (2017)
*Crocus sativus L.* extract containing polyphenols	*Crocus sativus L*	ALT, AST, ALP↓; CAT, SOD↑, MDA↓; TNF-α↓	decrease the levels of hepatic enzyme and proinflammatory cytokines markers in the INH and RFP-induced rats Wali et al. (2020)
Water extract from persimmon leaves	*Diospyros kaki Thunb*	ALT, AST, ALP, LDH, TBA, TBIL, DBIL↓; TNF-α, IL-6, IL-10↓; SOD, GSH-Px, GSH↑, MDA, NO↓	Enhance the body’s antioxidant capacity, scavenge oxygen free radicals, reduce inflammation Wang J et al. (2020)

Notes: INH, isoniazid; RFP, rifampicin; MDA, malonic dialdehyde; SOD, superoxide dismutase; GSH, glutathione; CYP2E1, cytochrome P2E1; TBA, total bile acid; DBIL, direct bilirubin; TBIL, total bilirubin; GSH-Px, glutathione peroxidase; CAT, catalase; TG: triglyceride; SIRT1, silent information regulator 1; PPAR-γ, peroxisome proliferator-activated receptor-γ; PGC1α, peroxisome proliferator activated receptor-gamma coactivator 1α; ROS, reactive oxygen species; NO, nitric oxide.

**FIGURE 4:**
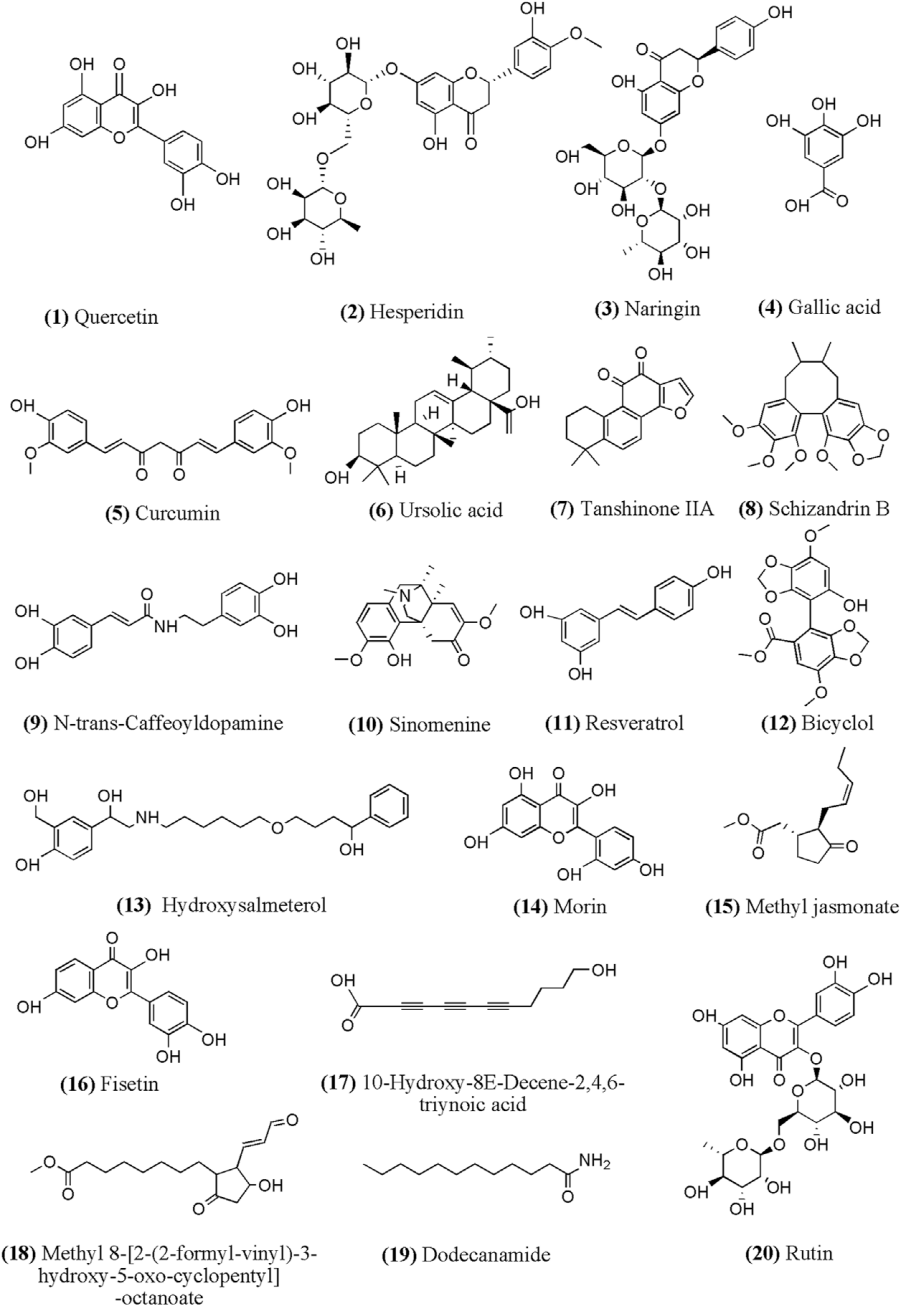
Chemical structural of part monomer compounds against INH/RFP-induced liver injury. Notes: (12)-(20) identified from *Crocus sativus L.* extract containing polyphenols.

According to related researches in recent years, we summarized the chemical structure of part monomer compounds in natural medicine against INH/RFP-induced liver injury in Figure 4.

## 6 Future research perspective

Liver injury is the most common adverse reaction of antitubercular drugs, and it is one of the reasons for the discontinuation of the treatment. Thus, solutions should be discovered to reduce and avoid liver injury. Some studies have found that INH-induced liver injury is dose-dependent. At a daily dose >0.3 g, the incidence of liver injury increases by 7.48%–15.48% for every 0.1 g increase in the daily dose (Yang et al., 2017). Therefore, establishing the dose-response relationship for antitubercular drugs in a population will help to individualize the dose, increasing their clinical safety.

INH and RFP-induced liver injury results from the intertwined action of multiple targets and pathways. The pathogenesis of liver injury is complex and has not been fully elucidated. Early studies on INH and RFP-induced liver injury were mainly focused on the apoptosis and necrosis of hepatocytes. Recently, pyroptosis and ferroptosis of hepatocytes were reported in pathological processes such as tissue damage and organ failure (Wang Y et al., 2021). The elucidation of cell death provides starting leads for studying the mechanisms involved in INH and RFP-induced liver injury.

Currently, the biomarkers for the detection and diagnosis of liver injury are still conventional liver function indicators (ALT, AST, and ALP). The dysregulation of these markers can also be caused by other factors unrelated to liver injury. Thus, these indicators are not good enough for accurate and sensitive prediction of liver injury. It has been reported that glutamate dehydrogenase (GLDH) in liver mitochondria is closely related to ALT levels. GLDH is liver-specific and can be a diagnostic indicator for DILI (Church et al., 2019). Furthermore, mitochondrial dysfunction has been implicated in the development of ATB-ILI. It is worth noting that elevated mitochondrial DNA (mtDNA) content was considered an independent determinant of ATB-ILI. Changes in leukocyte mtDNA may be used as a novel, specific ATB-ILI biomarker (Udomsinprasert et al., 2022). Since the pathogenesis of INH and RFP-induced liver injury involves multiple pathways, a single biomarker is insufficient to diagnose the liver injury. Other markers with higher specificity and sensitivity should be sought to facilitate the prevention and early detection of ATB-ILI.

The evaluation of INH and RFP-induced liver injury was mostly carried out in rats and mice. The results from different animal models are very diverse, which might be due to differences in drug tolerance in different animal strains. Unfortunately, there are currently no animal models available that could recapitulate the clinical manifestations of ATB-ILI for elucidating the underlying pathogenesis in detail. Compared with the traditional model animals, zebrafish have the advantages of small size, strong reproductive ability, simple mode of administration, short experimental period, highly conservative with human genes, and replicated diseases are highly similar to those in humans (Zhou, 2017). Jia et al. (2019) used the zebrafish model to explore the mechanism of INH-induced liver injury *via* ERS. Therefore, Zebrafish may be one of the potential models for evaluating DILI in the future.

Furthermore, traditional *in vitro* cell models gradually lose liver-specific functions during the culture process, and the dosage of the drug used in these studies is much higher than the actual clinical dose. Thus, using cell models has certain limitations in evaluating DILI. Some studies (Lu et al., 2017) have proved that INH did not cause cytotoxicity until 1 mmol/L in the 2D model; however, it showed cytotoxic effects in 3D HepaRG multicellular polyspheroid model (IC_50_, 700 umol/L). 3D cell models have certain advantages in simulating the *in vivo* cellular microenvironment and *in vitro* assembly of hepatocytes (He et al., 2021). Thus, 3D models should be used as *in vitro* models to evaluate DILI. Natural medicinal ingredients are important in preventing and treating INH and RFP-induced liver injury.

Natural medicinal ingredients have unique advantages in improving patients’ symptoms, reducing the risk of liver injury, delaying the disease’s progression, and enhancing the body’s ability to repair independently. The pharmacological mechanisms of natural medicinal ingredients and active ingredients are complex. The protective effect of natural medicinal ingredients on INH and RFP-induced liver injury involve regulating multiple pathways. Therefore, it is necessary to use modern scientific and technological means combined with theoretical knowledge of pharmacology, bioinformatics and so on to reveal further the mechanism, targets and the relationship between pathways of natural medicinal ingredients in the prevention and treatment of the INH and RFP-induced liver injury. Polysaccharides have unique advantages in treating INH and RFP-induced liver injury. The chemistry of polysaccharides is complex. Thus, purifying and identifying the composition and establishing the structure-activity relationship is necessary to provide stronger clinical evidence for anti-INH and RFP-induced liver injury. In addition, flavonoids also play a critical role in the treatment of INH and RFP-induced liver injury. However, flavonoids have both antioxidant and pro-oxidative effects. High-dose quercetin (1,500 mg/kg and 2000 mg/kg) may act as a pro-oxidant and cause oxidative stress (Singh et al., 2022). It is necessary to be aware of the side effects caused by flavonoids.

Intestinal flora disorder is closely related to liver diseases. According to the liver and spleen theory recorded in “Jingui Yaolue,” see the disease of the liver, know that the liver transmits to the spleen, and liver diseases could be treated by reinforcing the spleen (Zhao et al., 2016). It might become a new research direction to explore the protective effect of the interaction of natural medicinal ingredients and intestinal flora on liver injury caused by INH and RFP from the point of view of the liver-intestinal axis. Natural medicinal ingredients have the advantage of increasing efficiency and reducing toxicity, which can not only reduce the toxic and side effects of drugs but also strengthen the body and dispel pathogenic factors and enhance the immunity of patients to improve the curative effect. The evidence (Barua and Buragohain, 2021) suggested that curcumin nanoparticles inhibit the growth of the *Mycobacterium tuberculosis* H37Rv strain in mice and accelerate the clearance of *Mycobacterium tuberculosis* from the lungs of BALB/c mice by promoting an antitubercular response, thereby shortening treatment time. It also can restore INH-induced suppression of antigen-specific cytokines and the proliferation of T cells. It also reduces ATB-ILI in mice, enhancing their efficacy and reducing toxicity. Therefore, it might be a new perspective for future research to explore the therapeutic advantages of natural medicinal ingredients to improve efficacy and reduce the toxicity of conventional antitubercular drugs.

**FIGURE 5:**
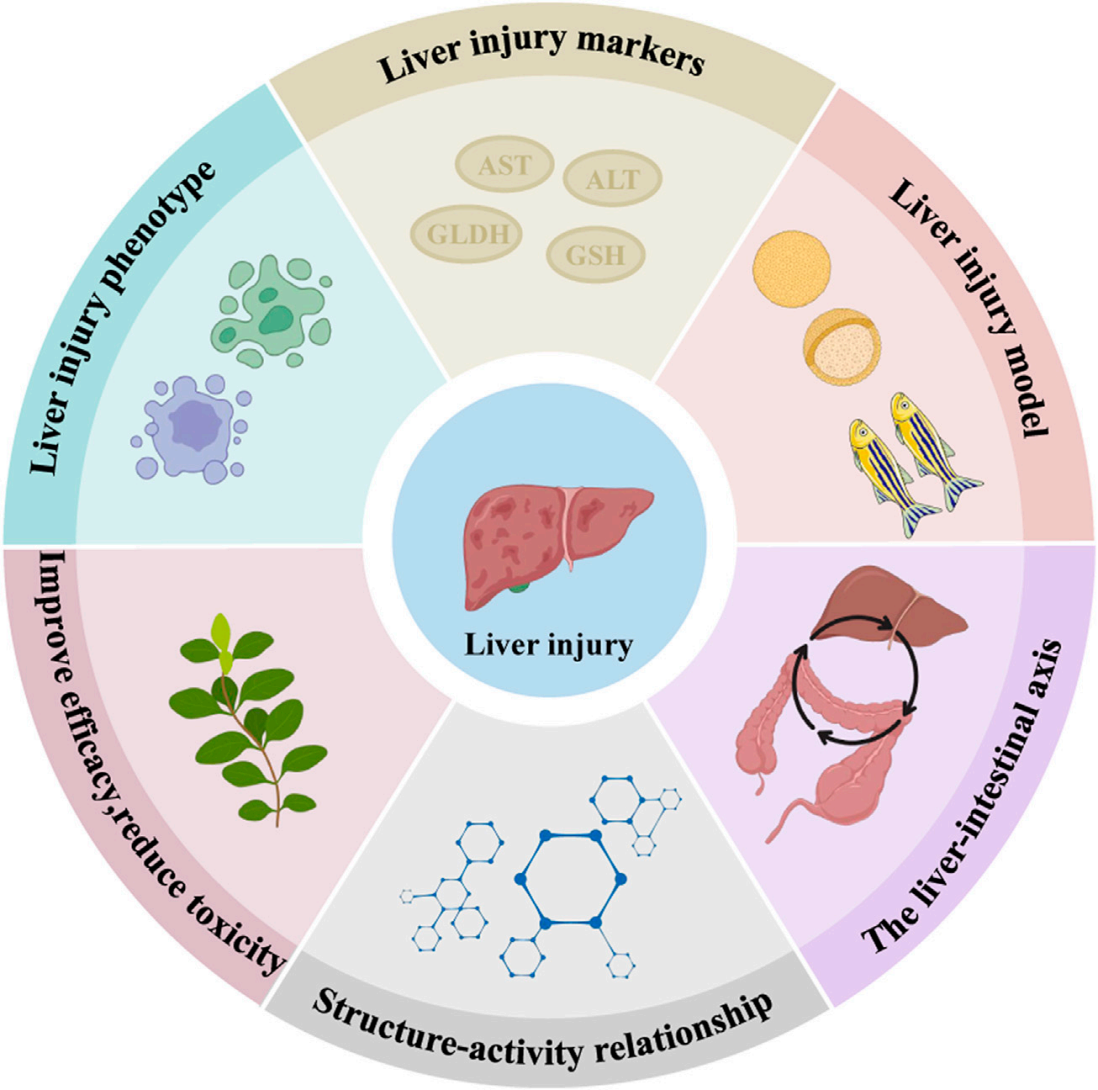
Predicted directions for later studies of INH and RFP-induced liver injury and the effects of natural medicinal ingredients. We predicted that the following six aspects might be studied in the future: Liver injury phenotype, liver injury markers, liver injury model, the liver-intestine axis, structure-activity relationship and improve efficacy, reduce toxicity.

This paper systematically summarised the recent literature on the molecular mechanisms of INH and RFP-induced liver injury. The protective effect of natural medicinal ingredients and its bioactive compounds on INH and RFP-induced livery injury were summarised. Thus, this review paper will be a valuable resource for understanding the molecular mechanism involved in INH and RFP-induced livery injury and the role of natural medicinal ingredients in protecting liver tissue. Further research should be carried out to discover novel solutions for preventing and treating INH and RFP-induced liver injury and the detailed molecular mechanisms involved in natural medicinal ingredients’ protective effect. Also, research on liver injury phenotype, identifying novel biomarkers of DILI and developing experimental models (*in vitro* and *in vivo*) should be carried out. The research on the liver-intestine axis and the structure-activity relationship should be further strengthened to enhance efficacy and reduce the toxicity of INH and RFP (Figure 5).
